# Mining privacy-preserving association rules using transaction hewer allocator and facile hash algorithm in multi-cloud environments

**DOI:** 10.1016/j.mex.2025.103317

**Published:** 2025-04-17

**Authors:** D. Dhinakaran, S. Gopalakrishnan, D. Selvaraj, M.S. Girija, G. Prabaharan

**Affiliations:** a Department of Computer Science and Engineering, Vel Tech Rangarajan Dr. Sagunthala R&D Institute of Science and Technology, Chennai, India; b Department of Computer Science & Engineering (Data Science), Madanapalle Institute of Technology & Science, Andhra Pradesh, India; c Department of Electronics and Communication Engineering, Panimalar Engineering College, Chennai, India; d Department of Computer Science and Design, R.M.K. Engineering College, Chennai, India

**Keywords:** Association rule mining, Distributed data analysis, Data encryption, Data privacy, Frequent itemset mining, Horizontal partitioning, Facile Hash Algorithm, Transaction Hewer and Allocator, and ATid algorithm

## Abstract

In this era of data-driven decision-making, it is important to securely and efficiently extract knowledge from distributed datasets. However, in outsourced data for tasks like frequent itemset mining, privacy is an important issue. The difficulty is to secure sensitive data while delivering the insights of the data. First, this paper proposes a new multi-cloud approach to preserve privacy, which includes two main components, named the Transaction Hewer and Allocator module and the Facile Hash Algorithm (FHA), in extracting the frequent itemset. All these components work together to protect the privacy of the data, wherever it is, during the transmission phase or the computation phase, even if it is raw data or processed data, on the different distributed cloud platforms. The complexities involved in the mining of frequent itemsets led us to introduce the Apriori with Tid Reduction (ATid) algorithm considering scalability and computational operational improvements to the mining process due to the Tid Reduction concept. We conduct performance evaluation on several datasets and show that our proposed framework achieves higher performance than existing methods, and encryption and decryption processes reduce the computational time by up to 25 % compared to the best alternative. It also exhibits approximately 15 % reduction in communication costs and displays scalability with the growing number of transactions, compared to the state-of-the-art evaluation metrics that indicate improved communication overhead.

• Introduces a multi-cloud privacy framework with Facile Hash Algorithm and Transaction Hewer and Allocator.
• Enhances scalability using ATid algorithm with Tid Reduction.

## Graphical abstract

**Figure:**
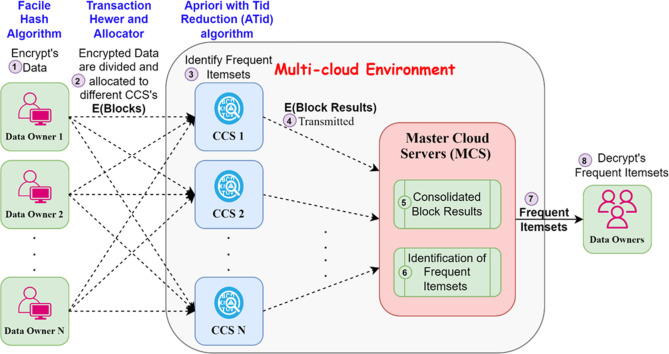

Specifications table

**Table:** 

Subject area:	Engineering
More specific subject area:	Privacy Preserving Association Rule Mining - to secure sensitive data while delivering the insights of the data.
Name of your method:	Facile Hash Algorithm, Transaction Hewer and Allocator, and ATid algorithm
Name and reference of original method:	Association rule mining, Distributed data analysis, Data encryption, Data privacy, Frequent itemset mining, Horizontal partitioning.
Resource availability:	N.A.

## Background

### Introduction

Data mining is defined as a process of discovering new, unexplored, may be valuable, non-trivial, significant trends or information from very large set of data [1]. Distributed data mining follows, as advanced networking technologies have enabled huge amounts of data to be collected and exchanged [2]. Conversely, technology has played a major role in highlighting data in adverse light. So, researchers are searching for ways to keep collected data private. Naturally, the reek of danger and cost of exposure centers around the data-mining application. If publicly available, the data would be useful for lots of research. Nevertheless, numerous persons or organizations are unwilling to share their records for information extraction because of privacy concerns, resulting in an information insufficiency and erroneous outcomes. Integrated data mining, and also cloud computer are capable of attaining rapid and related provisions of the vast amount of important information preserved in the major quantities of daily data that will be usually incomprehensible [[3], [4]–5]. When it comes to working with massive amounts of the data set, cloud computing acts as a solid (and scalable) framework in terms of either storage or processing power. Find helpful information and new knowledge when the combination of cloud computing and data mining [6]. But how its intimacy threats people’s privacy. On the other hand, the consequences of not factoring privacy between data mining can be devastating. How one might act on data largely dictates its value economically. Data analysis is to reveal news knowledge, draw conclusions, and improve actions or processes [7]. But if other people gain that information, they could employ it in negative manners and undermine these good intentions. That is a way big trouble for both the customer and cloud provider. One of the most severe problems is data confidentiality or data privacy [8].

A critical data privacy issue arises if the cloud provider misuses the data or information because the cloud provider holds all of the data. Similarly, if a cloud attacker or opposing authority gains illegal access to the cloud storage, they can mine the data to pull plenty of confidential information. There exist different approaches of data analysis or algorithms that derive valuable data from massive datasets by analysing behavioral and statistical data. The problem is that many cloud providers offer their customers data mining services themselves, which an attacker may leverage. Data can be protected in many different ways to prevent it from being stolen, accessed, or modified by an unauthorized individual. That includes encrypting the data. Encryption is often used as an additional level of mitigating controls over security rather than a separate layer. These functions mark one of the most significant interdisciplinary advancements in information technology, with the integration of cryptographic techniques with the knowledge discovery process [[9], [10]–11]. The privacy of the users and the entire database is secured using the cryptographical procedures. These approaches have been well-studied in a distributed setting. This requires data mining to involve data management that preserves confidentiality and privacy obligations.

#### Motivation

*Increase in Privacy Attacks in Distributed Data Mining:* The proliferation of multi-cloud environments for data sharing and analysis has also brought increased privacy challenges in ensuring confidentiality of sensitive data. Hence, organizations need to implement a mechanism to protect the proprietary data from unauthorized access while performing the frequent itemset mining without affecting the quality of the analysis.

• *Need for Collaborative Knowledge Extraction:* The demand for data and knowledge extraction for better insights has increased among different organizations, as they continue to share their data amongst themselves across multi-cloud platforms. But due to the sensitive nature of such data, secure and privacy-preserving techniques are crucial to enable such collaborations.
• *Existing privacy-preserving solutions:* The state-of-the-art privacy-preserving algorithms often have high overhead in computation complexity, communication cost and scalability avenues. These limitations severely restrict their applicability, notably in any real-time or large-scale application, such as multi-cloud data mining.
• *Security Challenges in Multi-Cloud Architectures:* With the increasing popularity of multi-cloud environments, the increasing complexity of securing data in transit and while processing is expanding. Existing approaches fall short when protecting data against cloud-based attacks, including unauthorized access, frequency analysis attacks, and information leakage during aggregation.

#### Objectives

• *Develop an All-Encompassing Privacy-Preserving Framework:* To develop a strong multi-cloud structure that maintains the secrecy of both unrefined and processed data, making sure that safely sensitive information is being protected when transitioned and mined without interrupting the mining process.
• *Improving Compactness:* Improve the frequency of analysis of transactions for frequent itemset mining by combining optimization plan algorithms adding the Pruning Algorithm and Tid Reduction (ATid) algorithm for scalability and minimizing computational cost.
• *Implement Strong Security from Attacks:* To provide defense mechanisms to counter common threats of multiple clouds (unauthorized data access, frequency analysis attacks, user data exposure, etc.) using advanced concepts like the Transaction Hewer & Allocator (THA) as well as the Facile Hash Algorithm (FHA).
• *Validate Performance Metrics:* To meticulously test the proposed model framework with real-world datasets and application to key performance metrics such as computational time, communication overhead and scalability which ensures the system is scalable when there is exponential growth of data and transactions.
• *Foster Secure Collaboration Across Organizations:* Allowing organizations to securely exchange knowledge from distributed datasets without exposing any sensitive data enabling collaboration across organizations with controls over data privacy.

The rest of this work is organized as follows: A short background on relevant work is given in Section 2, pointing out the limitations of current privacy-preserving frequent itemset mining methods. In Section 3, we present the proposed model, including the working principles of the Facile Hash Algorithm (FHA) and the Transaction Hewer and Allocator (THA) algorithm, two algorithmns that we consider the core of our privacy framework. In Section 4, we describe our mining approach making use of the Apriori with Tid Reduction (ATid) algorithm, which provides efficient and scalable mining of frequent itemsets. Section 5 presents results, including comparisons with traditional and privacy-preserving algorithms, as well as a comprehensive analysis of privacy and security. The study ends with Section 6 where the contributions are summarized and future directions are outlined.

### Related works

In the current data-centric world, Privacy-preserving Association Rule Mining (ARM) techniques have become one of the major tools for improving safety. This includes a wide range of techniques such as secure multiparty computation, anonymization, cryptographic methods, or obfuscation to protect sensitive data [12]. Although these methods provide substantial benefits concerning privacy protection, they also have disadvantages that need to be taken into consideration for solving the challenges related to data security and privacy preservation. Nonetheless, despite the effectiveness of privacy-preserving ARM techniques in reducing privacy threats, a number of limitations remain. One major challenge is data analysis correctness decay over time especially when new versions of the same dataset become available [13]. The results of such analysis, however, become progressively more difficult to maintain correctly as the underlying data change and risk being out of date in very short order, and potentially leading to different people having different results on the same data if they run the analysis at different times. Furthermore, privacy-preserving ARM methods frequently have higher computational overheads compared to traditional data analysis methods, yielding less efficient execution and scalability. Such inefficiencies hinder their practical realizability and widely spontaneous deployment into the real world.

Moreover, cryptographic methodologies are commonly used in privacy-preserving ARM, introducing additional privacy risks under contracted cloud services contexts [14]. However, due to the absence of a genuinely reliable third party, data privacy and security are often questioned, since a third party may have ulterior motives or may eventually be misused. In addition, the complexity of cryptographic protocols could introduce vulnerabilities that may be exploited by malicious actors, amplifying privacy threats and diminishing the efficacy of ARM techniques that preserve privacy. Although they offer privacy, current ARM methods suffer from a few limitations that limit their performance and practical use. These limitations include:

• Accuracy of data analysis deteriorating over time, especially with the updated datasets.
• Higher computational expenses and lower efficiency than traditional data analysis ways.
• Cryptographic methodologies (especially in contracted cloud services) privacy risks.
• Absence of an authentically trusted third party to regulate data privacy and safety
• Flaws in cryptographic protocols exploitable by bad actors.

Working on these drawbacks is imperative to building more fortified and efficient privacy-preserving ARM approaches that can fulfil the dynamic requirements of data security and confidentiality attention in contemporary data-driven society. Giannotti et al. Within the realm of corporate privacy-preserving architectures, [15] proposed a set of ARM outsourcing tasks. Although this method provides strong privacy assurances, its scalability may be limited for large datasets. Finally, background knowledge-based attack models used in existing methods can make those methods less resilient to attacks in real-world dynamic environments as the knowledge of the adversaries change over time. Pika et al. [16] addressed healthcare privacy by investigating anonymization methods and their effect on process mining results. Although their method for privacy metadata achieves a good result for tracking all the modifications in records, this may not successfully work for cases where an anonymization approach could result in loss of information and distortion and ultimately utility of the results that are mined.

Wenbo et al. [17] proposed a multi-characteristic repeal auction scheme based on oblivious transfer and anonymization techniques based on the Paillier cryptosystem. Although their technique provides robust privacy guarantees, using a set of servers for performing the computations adds complexity and possible failure points, affecting the system reliability and performance. Dong et al. [18] has proposed a local linear regression with privacy protection framework, which is based on LWLR algorithm. Although their methodology ensures good protection of user privacy via encryption, it has some computational overheads and efficiency concerns, especially in large datasets or more complex regression models. Zhang et al. [19] proposed an efficient MASK algorithm for ARM that drives bit-and operations at the PBEK level. Their method can lead to better computational efficiency, which induces a lack of accuracy even in situations where the distributions of data are not homogeneous or itemsets are scarce. Manasi et al. [20] focused on the approach for DO was based on creating horizontal part of the database that is split into several segments and rules that define how the second part is filtered in order to protect sensitive information. Although their method has strong privacy guarantees, it could struggle in contexts where the resulting rules are very strict, therefore, providing limited utility of the mined results.

Shuo et al. [21] implemented a homomorphic encryption framework ensuring confidentiality in frequent itemset mining on public cloud services. Although their method securely maintains the privacy standards of data, it could still be affected by performance overheads or scalability issues, especially in mining tasks that require more resources or datasets that are larger in size. Ahmed et al. [22] proposed utilizing Association Rules to analyze medical datasets at hospitals without sending the data externally. The utility of their method is reduced in cases where collaborative analysis or combination with external datasets is necessary to get a full picture, even though their mitigation reduces the privacy risk from transferring data. Hongping et al. [23] a cryptosystem for privacy-preserving ARM to twin-cloud designs. Their solution provides better scalability and data diversity with multi-user public key management, but is challenged with heterogeneous data sources or dissimilar encryption policies. Chenyang et al. [24] proposed an efficient protocol to compute the frequency of item sets with anonymity. They also implemented a blocking mechanism to improve mining efficiency. So, its pet project is splitting encrypted transactions into blocks, reducing the overall cost of computing them for the purpose of mining. But there could be some restrictions on situations where the blocking technique incurs an overhead or gets into the demand on mined results.

Kenta et al. [25] indeed addressed these needs, working with private-set intersection methods to present a privacy-preserving mechanism that allows organizations to conduct association rule mining out of vertically partitioned data. Their method allows for sharing data while maintaining confidentiality between organizations. Though useful, this method may lead to several issues, including problems regarding scalability and increased computational complexity, especially in datasets that are large in nature or with extensive privacy requirements. Tsou et al. [26] focused on participant methods used different candidate itemsets by using multiple support threshold, DPARM algorithm. Using uniform partitioning to reduce dataset dimensionality, they achieve effective management of privacy risks alongside mining efficiency. Their approach might be inefficient for some datasets, as its efficiency depends on the distribution and characteristics of the dataset. Qilong et al. [27] proposed a method to ensure parking for finding association rules in distributed databases in terms of differential privacy. It works by aggregating data on an intermediary server while not considering the security of that server. HC and MS mining methods are the two techniques they suggested for various attack models of association rule mining. Although their proposal provides strong privacy guarantees, it may run into some issues regarding scalability and performance, particularly when it comes to resource heavy mining tasks or heterogeneous datasets.

Nikunj et al. [28] concentrated on privacy-preserving ARM over vertically fragmented healthcare data. They split the healthcare data vertically among three systems, namely Key generation center (KGC), Participant-A (patient health data), and Participant B (Out-patient Records). Although this method overcomes privacy issues connected with sharing healthcare informations, it may have to contend with difficulties grounded in combining various formats of data from different sources. This work dealt with the ARM in a dispersed context, leveraging a semi-trusted intermediary server. Thus, the server and the participant cannot see the private transactions of other participants. To address these challenges, we proposed two algorithms for privacy-preserving ARM that are less communication time and computation cost, efficient data utilization, performance, privacy preservation, and exact results. In reviewing the existing literature on privacy-preserving ARM techniques, it is likely to notice that large amounts of work have been done in handling the data security and privacy preservation issues in many areas. By utilizing techniques such as secure multiparty computation, anonymization, cryptographic approaches, obfuscation, etc., privacy-preserving ARM techniques reflect their significant advantages to balance security and usability when addressing privacy concerns. Nonetheless, current methods often suffer from limitations such as degradation of correctness of results over time, high computational costs as well as accompanying privacy risks with crypto-based approaches.

Compared to existing approaches, our novel solution overcomes these drawbacks and provides multiple other key benefits. First, we provide a unique two-algorithm for amicable item-set mining in a multi-cloud context, which uses a Transaction Shearer and Allocator algorithm and a Facile Hash Algorithm. This design allows for the secure sharing of data by the participants without the loss of confidentiality and reduces privacy risks in contracted cloud services. Furthermore, we utilize Apriori with Tid Reduction (ATid) inside our framework to speed up the computation process and be scalable and feasible due to fewer transactions are being examined. Moreover, different from prior approaches which potentially compromise correctness of the analyzed data over time, our method ensures integrity and utility of the analyzed data by all means through keeping the raw data and processed data components secret. Our work overcomes several of the limitations of existing approaches—making a significant contribution to the development of privacy-preserving ARM techniques with stronger privacy guarantees, as well as being scalable and efficient in a multi-cloud environment.

## Method details

**Fig. 1:**
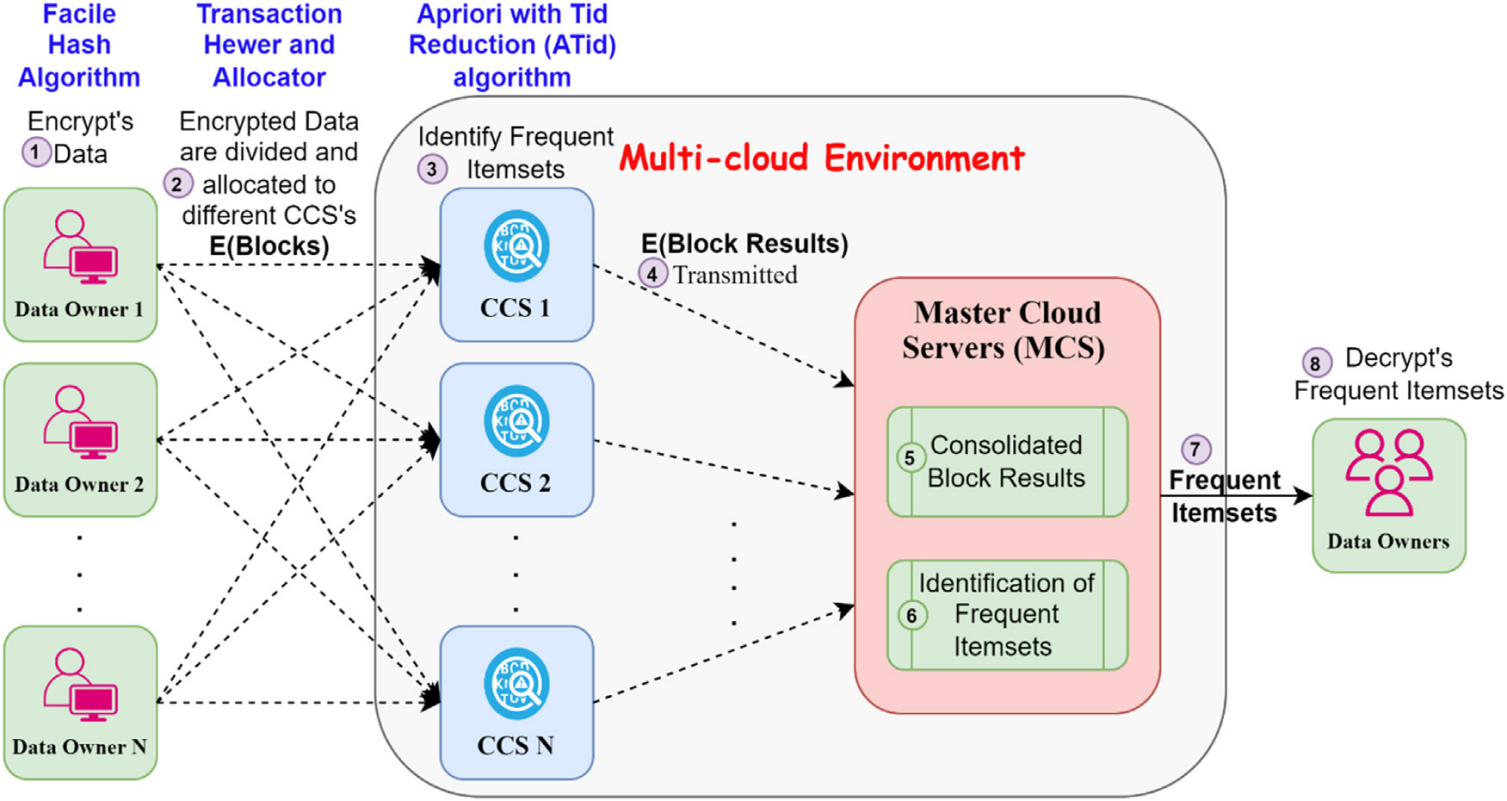
System Model.

In our architecture, we maintain very high privacy protection level in the Process of ARM in distributed environment. We understand that data privacy is a concern for data owners, hence we have modelled our approach in such a manner that treats data of all owners privately during the collaborative data mining process. Each data owner performs preprocessing prior to cloud transmission in order to preserve data confidentiality. The model is built around the concept of three general parts, which are Data Owners (DO), Computing Cloud Servers (CCS), and Master Cloud Servers (MCS), as demonstrated in Fig. 1. First, the data owner protects its personal data against CCS and MCS by encoding his/her data with Facile Hash Algorithm (FHA). Our Facile Hash Algorithm has both a one-way function and an unknown cryptography method that guarantees extreme anonymity. Also, relative to currently available cryptographic algorithms such as the secure hash algorithm and MD5 the Facile Hash Algorithm consumes less computational time and costs.

These secured datasets are then split and deployed across distinct clouds via the Transaction Hewer and Allocator (THA) technique. The THA algorithm mainly consists of two functions, i.e., the data partitioner, which partitions the encrypted dataset, and the partition allocator, which allocates partitioned blocks to the respective clouds. Then, each cloud computing server applies the ATid algorithm to mine frequent itemsets from the sub-blocks it receives. ATid algorithm which is a fundamental improvement based on Apriori algorithm, to improve the efficiency of the algorithm, not only introduced for Tid Reduction. It generates candidates of size 1, and then iteratively joins candidates to find infinitesimal itemsets of size n. Tid Reduction is used in this process, which iteratively removes less frequent transactions, yielding a significant drop in the number of transactions passed to support counting in subsequent steps. The Apriori algorithm leverages this to prune the candidate set based on the significantly reduced transaction list, maximizing its efficiency [29].

After the periodic itemsets are evaluated by each cloud computing server from their sub-blocks, the corresponding results are sent to the Master Cloud Server (MCS). At last, the MCS aggregates the block results of all cloud servers, tests out the final frequent itemsets, and outputs the results to all data owners. In an easier hash method, the information partitioner, the information allocator, and the information holders’ keys are three variables that preserve real communication in gathering frequent element sets. In the case of the simple hash, the chaining variable changes when the key changes, resulting in mixed results. As for the partition function, the partitioner distributes the data owner dataset with respect to the cloud size, then sends the partitioned dataset (block) to the cloud randomly via the data allocator. While embedding the ATid algorithm to our architecture adds depth and efficiency in the multiple of privacy-preserving ARM solution, hence equipping it with the capability to obtain frequent itemsets with a higher degree of accuracy and a timely identification with the addition to maintaining both the privacy and the security of the data that is being exchanged in the collaborative data mining process. Table 1 presents a summary of all symbols and definitions used in the proposed work.

**Table 1:** Notation and Meanings.

Notation	Explanation
K[i] and K[r]	Constants
D	Input Dataset
K	Key
$A_{K}$	ASCII Key
$B_{K}$	Binary Key
$B_{d}$	Binary Numbers
$S_{0}$ to $S_{7}$	Sub-Blocks
$F_{O}$	Final Output
$H_{C}$	Hash Code
$P_{K}$	Partitioner Keys
Hs	Hash Space
S	Shares
Th	Threshold for Itemset Sizes
$d_{t}$	Data Transaction
BI	Block Index
$B_{j}$	Data Blocks
F	Frequent Itemsets
$T_{id}$	Transactions

## Facile hash algorithm

Facile Hash Algorithm (FHA) aims to provide status and hash chain updates at an unprecedented level for access control protocol when it comes to data privacy and security of data in distributed environments for either research on machine learning or a cloud computing security vulnerability. FHA, as a vital part of privacy-preserving Association Rule Mining (ARM) systems, not only assists IL without compromising the raw data but also greatly improves the ability to process and analyze data. The FHA, a unique hashing algorithm, incorporates a robust one-way function that generates a unique 16-bit block of hash code (HC) for every input dataset (D) of a maximum 32-bit value. FHA is different from other hashing algorithms in that it produces a uniformly distributed random-like output across datasets. Furthermore, any alteration in the input data set is highly likely to produce a different hash code, and this guarantees the integrity and privacy of data.

### Working principles of FHA

The steps that are used to operate the Facile Hash Algorithm are precisely designed to generate secure and privacy-friendly hash code from the input stream. First, a data owner chooses a key for initiating the FHA process, the key can be of 8 bits alphanumeric characters. Then it changes the key to its underlying ASCII code, which gives a 64-bit binary string. That binary representation is then passed through a reducer algorithm which yields a 32-bit binary number. The Facile Hash Algorithm (FHA) is a process divided into a set of steps to secure and effectively hash data. First, the input string will go string padding (i.e. put zeros so that it becomes a 32bit string). Then the string gets split into individual characters (single input bit), then ASCII conversion (each character to its ASCII code). This decimal number is then converted into binary number, leading to a final 256-bit output. These binary data is split into eight blocks of 32 bits and operated on via right shifts ($S_{0}$ through $S_{7}$). It is a round algorithm consisting of 4 rounds which uses a 256-bit block, ABCD variables, K[i],K[r] constants. Then, in the last step, the result of the fourth round is compressed to 128 bits using a reducer algorithm. The binary result is then turned into ASCII code, whereby an eight-bit segment is replaced by alphanumeric digits.

The benefits derived from FHA make it very useful for privacy-preserving Association Rule Mining (ARM) in distributed systems. Extreme anonymity guarantees that any variation at the input level leads to a huge variation at the hash code level to keep data secure. The efficient computation of the algorithm means that a lot of time is saved in generating the hash codes therefore cutting computational overhead. Also, FHA improves data protection by making raw data anonymized by using encryption so as to minimize the probability of an offender getting access to the information. Apart from privacy-preserving ARM, FHA has uses in other disciplines that seek to securely preserve their data. It assumes the crucial role in constructing data security applications including secure communications protocols and data integrity check, as well as controlling data access. In blockchain technology, FHA plays a crucial role of hashing transactions with a view of enhancing the ledger’s security and measurably. It also serves in digital forensics since it can arrive at hash codes for specifically applied digital evidence in a bid to help in making certain that the data has not been altered. The Facile Hash Algorithm is a wonderful improvement in hashing manners that provides optimum standards in data security and absorption. Due to this simple one-way function and structure, it is a wonderful asset to privacy-preserving ARM systems and many others. With concern to security and privacy requirements for Data processing, FHA is in a position to offer great contribution in the protection of confidentiality of information and guarantee the authenticity of data transfers in global distributed system environments.

### Facile hash algorithm (FHA)

1. *Initialization:*
  • Let D denote the input dataset, where ∣D∣ ≤ 32 bits.
  • Let Key (K) represent the selected key for FHA initialization, with a length of 8 bits alphanumeric characters.
  • Let K[i] and K[r] denote constants used in the round process, where *i* = 0,1,2,3,4 and *r* = 4,3,2,1,0 respectively.
2. *Key Transformation:*
  • Convert the selected key K into ASCII code: ASCII Key $\left(A_{K}\right)$.
  • Convert $A_{K}$ into a 64-bit binary representation: Binary Key $\left(B_{K}\right)$.
  • Reduce $B_{K}$ to a 32-bit binary number using the reducer algorithm: Reduced Key.
3. *Dataset Transformation:*
  • Append zeros to the end of the dataset D to ensure a length of 32 bits if |*D*| < 32.
  • Break D into individual characters, each represented by a 1-bit binary value.
  • Convert each character into its ASCII code: ASCII Characters.
  • Convert ASCII codes into binary numbers, resulting in a 256-bit binary representation: *B_d_*.
4. *Block Division:*
  • Divide $B_{d}$ into eight blocks, each containing 32 bits:
    $M_{0}=Block_{1}$$M_{1}=Block_{2}$⋮
$M_{7}=Block_{8}$
5. *Shift Operation:*
  • Perform a shift operation for each sub-block from $S_{0}$ to $S_{7}$, resulting in modified sub-blocks:
    $M_{0}=S_{0}$$M_{1}=S_{1}$⋮
$M_{7}=S_{7}$
6. *Round Process:*
  • Execute four rounds of operations, each utilizing a 256-bit block, variable ABCD, and constants K[i] and K[r]:
    Round 1:
    ○ $ABCD_{1}=F_{1}\left(M_{2},M_{5},M_{4}\right)$
    ○ $F_{1}=\left(M_{2}⊕M_{5}\right)\wedge \left(M_{2}⊕M_{4}\right)$

Round 2:
  ○ $ABCD_{2}=F_{2}\left(M_{3},M_{4}\right)$
  ○ $F_{2}=\left(M_{3}\wedge M_{4}\right)⊕\left(\neg M_{4}\right)$
Round 3:
  ○ $ABCD_{3}=F_{3}\left(M_{2},M_{5},M_{3},M_{4}\right)$
  ○ $F_{3}=(M_{2}\wedge \left(\neg M_{5}\right)\wedge \left(M_{3}⊕M_{4}\right)$
Round 4:
  ○ $ABCD_{4}=F_{4}\left(M_{2},M_{5},M_{3},M_{4}\right)$
  ○ $F_{4}=\left(M_{2}\vee M_{5}\right)⊕\left(M_{3}\vee M_{5}\right)$

7. *Final Concluding Process:*
  • After the fourth round, obtain the output as a 256-bit block.
  • Use the reducer algorithm to convert the 256-bit output into a 128-bit representation: Final Output $\left(F_{O}\right)$.
  • Convert $F_{O}$ from binary to ASCII code, replacing each 8-bit segment with alphanumeric characters to obtain the hash code: $H_{C}$.

The Facile Hash Algorithm (FHA) operates through a series of mathematical transformations and operations on the input dataset and key. By formalizing the algorithm into a mathematical representation, we gain a deeper understanding of its inner workings and can more precisely analyze its properties and behavior.

### Transaction hewer and allocator algorithm

The Transaction Hewer and Allocator (THA) algorithm is crucial for efficient distribution of data among Computing Cloud Servers (CCS) based on a data allocation strategy. This algorithm, which is known as a horizontal data partitioning technique, takes the full responsibility for the CCS and partitions the whole data source. The THA algorithm uses the data partitioner to divide the information source using horizontal fragmentation. Horizontal fragmentation is where you split the data set into subsets or fragments based on some criteria or predicates concerning the characteristics of the data set. The example below shows fragments of an overall dataset sliced up using predicates on 1 or more relationship attributes. The data partitioner leverages the horizontal fragmentation to increase the versatility of the system, allows data colocation, and supports processing speed [30]. Complementing the data partitioner is a partition allocator feature that allocates data blocks to Computing Cloud Servers (CCS). It works by creating unrestricted block allocations as per the number of existing CCS (N). In addition, the partition allocator method accomplishes its allocation in O(1) time complexity for effective and fast data blocks distribution. Additionally, the partition allocator uses randomization to allocate blocks for load balancing and preventing possible data processing bottlenecks.

**Fig. 2:**
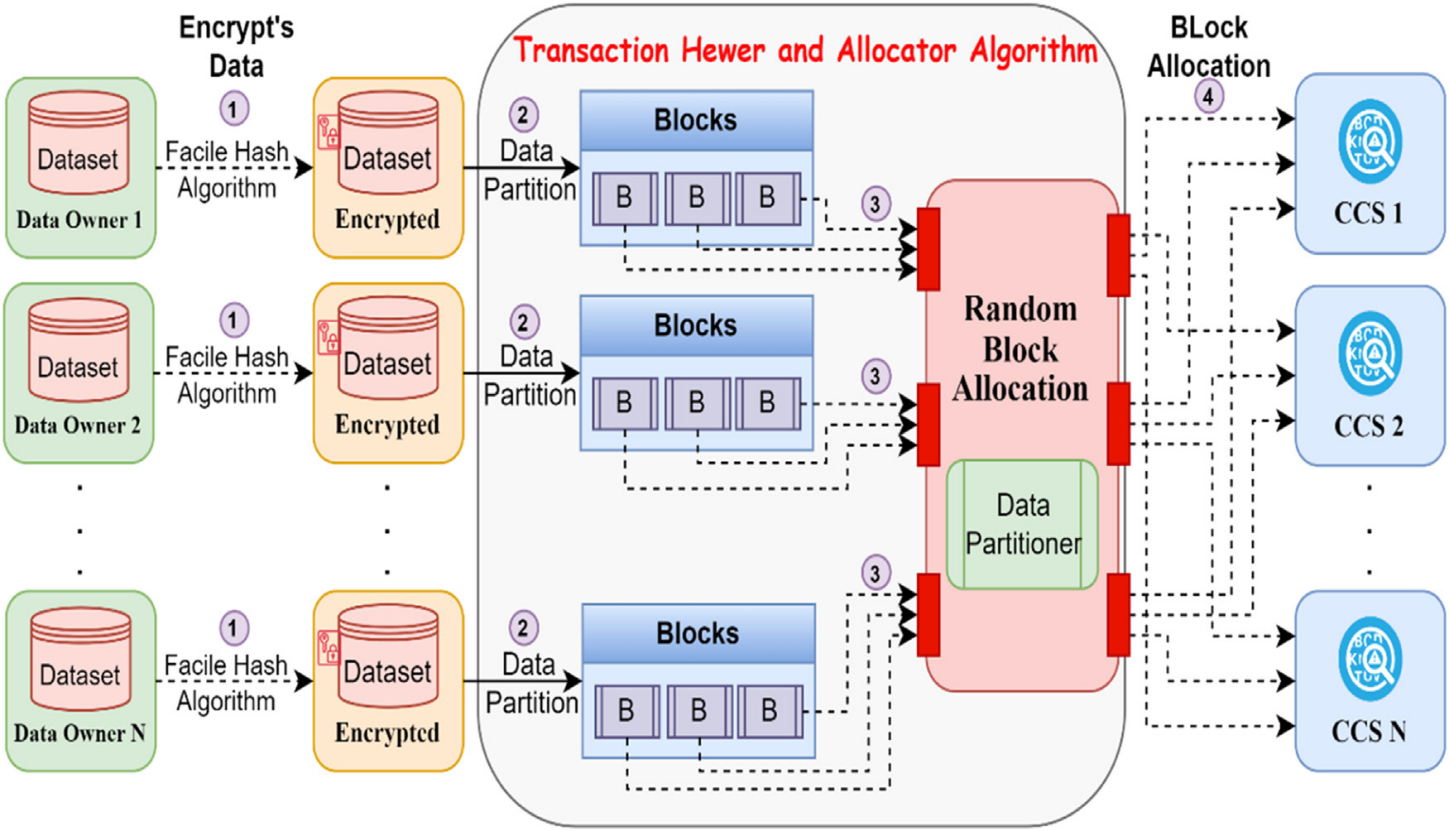
Working of Transaction Hewer and Allocator Algorithm.

Fig. 2 shows the schematic insight of the working of transaction hewer and allocator algorithm. THA algorithm works by use of data partitioner which helps to segment the full information source into small chunks or partition horizontally. A partition refers to a specific subset of the dataset that is defined by an attribute or predicate. In other words, they vertically separate the parts of the tables according to a horizontal partitioning process, which rearranges the distribution of the data, wherein Computing Cloud Servers (CCS) allocate relevant data portions based on certain pre-defined parameters. The partition allocator component then takes over and produces unbounded block allocations in relation to how many CCS (N). This allocation is performed at constant time complexity (O(1)), allowing for the quick and efficient assignment of data blocks to each CCS. Additionally, the partition allocator utilizes a random approach for block allocation, improving load balancing and ensuring a fair allocation of computational resources across the CCS infrastructure.

### Data partitioner

In the overall architecture, the Data Partitioner component (DPC), is responsible for distributing the dataset among the Computing Cloud Servers (CCS). It's use reliable hashing to distribute the data among the CCS as equally as possible while preventing the localization and hotspots. The Data Partitioner uses trusted hashing functions to allocate the dataset parts to individual CCS. All dataset entries are denoted by a unique Partitioner Key ($P_{K}$), randomly assigned by the data providers to avoid localization. The partitioner converts the $P_{K}\;$values into hash values and distributes the values into specific blocks. This guarantees a balanced distribution of data across CCS infrastructure and ensures that hotspots are less likely to develop; it does this through the use of consistent hashing algorithms. To provide an even distribution of itemsets among CCS, an equal chance is given to each CCS of receiving a data item's block value. The way it works is that the block value for a single CCS is chosen in proportion to the number of itemsets that are assigned to that CCS. Doing so guarantees that every CCS is given a fair chance of receiving data item blocks, regardless of how many itemsets it contains. The size of the key (K) assigned to each CCS is determined by the number of shares allocated to that CCS. As the number of shares (S) in a CCS increases, the key length assigned to that CCS also increases. This adjustment in key sizes ensures that each CCS receives an appropriate key size based on its share of the dataset, thereby optimizing data distribution and ensuring efficient data processing.

Steps:

• The block value (Block.val) assigned to each CCS is determined by taking the modulo of the $P_{K}$ values with the total number of CCS, ensuring uniform distribution.
• The probability of a block value belonging to a specific CCS is proportional to the number of shares (S) in that CCS.
• The size of the key (K) assigned to each CCS is directly proportional to the number of shares (S) in that CCS.
• The key space (K.CCS) for each CCS is calculated based on the threshold for itemset sizes and the size of the hash space (Hs) available.

The Data Partitioner component ensures efficient and equitable distribution of data among Computing Cloud Servers (CCS) by employing reliable hashing techniques and uniform distribution principles. By adjusting key sizes based on the number of shares allocated to each CCS, the Data Partitioner optimizes data distribution and promotes efficient data processing in multi-cloud environments.

*Block Value Calculation:*
  (1) $$\text{Block}.\text{val}\;=P_{K}\;\text{values}\;\%\;\text{Sum}\;\text{of}\;\text{CCS}$$

Eq. (1) determines the block value assigned to each CCS by taking the modulo of the Partitioner Keys (PK) with the total number of Computing Cloud Servers (CCS), ensuring a balanced distribution of data blocks.

Probability *of Block Value in a CCS:*

An identical quantity of itemsets is assigned to each CCS in a uniform distribution. According to (2), (3), each CCS should have an equal chance of being selected for a data item's block value whenever the amount of itemsets in each CCS is equal.

(2) $$Prob\;\left(Bv\in CCS_{1}\right)=Prob\;\left(Bv\in CCS_{2}\right)=Prob\;\left(Bv\in CCS_{3}\right)=\ldots$$

(3) $$\frac{Prob\left(Bv\;\in \;CCS_{1}\right)}{Prob\left(Bv\;\in \;CCS_{2}\right)}=\;\frac{S\left(CCS_{1}\right)}{S\left(CCS_{2}\right)}$$

*Adjustment of Key Sizes:*

Eqs. (4) and (5) illustrate that the size of the key (K) assigned to each Computing Cloud Server (CCS) is directly proportional to the number of shares (S) allocated to that CCS. As the number of shares increases, the key size also increases, ensuring efficient data distribution and processing.

(4) $$K\left(CCS_{1}\right)\;\alpha \;S\left(CCS_{1}\right)$$

(5) $$K\left(CCS_{2}\right)\;\alpha \;S\left(CCS_{2}\right)$$

*Key* Space *Calculation for CCS:*

Eqs. (6) and (7) calculates the key space (K.CCS) for each Computing Cloud Server (CCS), considering the threshold for itemset sizes (Th) and the available hash space (Hs). It ensures that each CCS receives an appropriate key space based on its share of the dataset and the overall distribution of keys.

(6) $$K_{s}=\;\frac{Th_{k}}{\sum_{a=1}^{n}Th_{a}}\;\left(100\right)$$

(7) $$K.\text{CC}S_{k}=\frac{K_{s}}{\sum_{a=1}^{n}K_{a}}\left(H_{s}\right)$$

Eqs. (1) to (7) provide a quantitative understanding of the key aspects of the Data Partitioner's operation, including block value calculation, probability distribution, adjustment of key sizes, and key space allocation.

*Partition Allocator*

The architecture consists of four primary components, namely the Partition Allocator, which serves a crucial role in data allocation as discussed below. These blocks are then distributed among the CCS's based on this algorithm such that data is as well planned as possible, leading to optimum resource usage and efficient data processing. The partition allocator simply loops through the blocks in the list, repeatedly shuffling them, and pops from the first. By performing random shuffling on the entire blocks, we can guarantee that every block has the same chance of being picked as the last element in the block list, which is 1/N with N being the total number of blocks. The partition allocator returns a sequence of blocks, uniformly shuffled. This chain directly corresponds to a tape of CCS, the rate of each CCS to get the chain is each CCS's working. As an example, most likely there is a column four blocks: B1, B2, B3 and B4. For example, the output sequence generated by the THA algorithm may be B3, B1, B4, and B2 (after shuffling). All these blocks are assigned to a corresponding CCS in order based on this shuffled sequence. E.g. CCS1: B3, CCS2: B1, CCS3: B4, CCS4: B2 With this allocation, data blocks are distributed evenly across the CCS infrastructure, avoiding data processing bottlenecks on specific servers. The partition allocator promotes better resource utilization and efficient data processing by ensuring that the data blocks are more or less evenly distributed among the CCS. By mitigating both bottleneck risks and providing efficient utilization of computational resources, the performance of the system is further optimized by this balanced allocation strategy.

### THA algorithm

*Data Partitioner Process:*

Input: $d_{t}$, $p_{k}$, N number of CCS

Output: A-List, L of N BI from 1 to N-1

1. Initialize:
  • Compute Block Code BC($p_{k}$) from the Key $p_{k}$.
  • Calculate BI as $BI=BC(p_{k})modN$.
  • Initialize an array SB to store data transactions for each Block Index.
2. Partition Data Transactions:
  • Assign the data transaction dt to the Block Index BI in the array SB.
3. Generate A-List:
  • Construct A-List L of N BI from 1 to *N* − 1 representing the partitioned data blocks.

*Partition Allocator (L) Process:*

Input: List L of N blocks, A-List

Output: Transformed A-List L of N blocks after allocation

1. Initialize:
  • Set *F* = *N* − 1, where F represents the maximum block index.
2. Allocate Blocks to CCS:
  For F to 1 do:
  ○ Generate a random integer r within the range of data transactions.
  ○ Swap the blocks Ls[dt] and Ls[r] in the list Ls.
  ○ If *r*=dt, the block remains unchanged.
3. Return Transformed A-List:
  • Return the transformed A-List L after allocation.

### Mining process

As shown in Fig. 4, in our proposed framework every Cloud Computing Server (CCS) has two key duties, to determine the block frequent itemset for the block assigned by the data owners (DOs) and to exchange the calculated block result with the Main Computing Server (MCS). Ensuring the integrity and reliability of digital evidence in cloud environment, is a process through which you need to follow. The CCS utilizes the ATid algorithm, which is an essential part of our framework, to determine the frequent itemset for the assigned block. This is what set our approach, called ATid, apart from classic methods that include Tid Reduction for enhanced performance and scalability, specifically tackling the issues surrounding data processing in a large-scale cloud way. The ATid algorithm employs a principle similar to that of the Apriori algorithm but aims to optimize the process with the addition of Tid Reduction. At the beginning, it finds all size 1 frequent itemsets to create the frequent itemset list (L) and transaction list ($Tid_{List}$). Then, at each iteration (k), it generates candidate itemsets of size *k* + 1 which are determined based on frequent itemsets size k.

The main novelty lies in the Tid Reduction step where the algorithm processes transactions, uncovering infrequent itemsets in each transaction. If the previous iteration's results classify all itemsets in a transaction as infrequent, the whole transaction is discarded from the next step ($Tid_{List}$). All this intelligent pruning already saves a lot of scanning of transactions to count support for these transactions in next steps. Even more, the algorithm prunes candidates in the reduced transaction list, it discards any candidate whose corresponding subset of transactions is an empty list, since it cannot be frequent. In the end, the support count is computed for the remaining candidate itemsets in the reduced transaction list and the frequent itemsets are determined for the current size (*k* + 1). The common clouds data sets have advantage from cloud environment at time when our ATid algorithm performs Tid Reduction it directly reduces the number of transactions taken for support counting in reducing the transaction taken in a data set hence it improves the overall working efficiency of the Apriori algorithm. Not only this optimization helps to expedite the identification of frequent itemsets, but also enhances the efficiency and scalability of our proposed framework.

*Support Count Calculation:*
  (8) $$Support\left(Item\right)=\frac{Count\;\left(\frac{Item}{Bj}\right)}{|Bj|}$$
*Minimum Support Threshold:*
  (9) {min_supt}×|Bj|
*Threshold Frequency for Data Block:*
  (10) $$\left\{min_{fj}\right\}\;=\;\left\{\text{min}supt\right\}\;\times \;\left|Bj\right|$$
*Condition for Frequent Itemset:*
  (11) {Support}(Item)<{minsupt}
*Condition for Frequent Itemset (Aggregate):*
  (12) $$=\sum_{j=1}^{n}Count\left(\frac{Item}{B_{j}}\right)<\sum_{j=1}^{n}min\_f_{j}$$
  (13) $$=\sum_{j=1}^{n}Count\left(\frac{Item}{B_{j}}\right)<\sum_{j=1}^{n}min\_B_{j}=\sum_{j=1}^{n}min\_supt*\left|B_{j}\right|$$

Eq. (8) calculates the traditional support count for an item in the entire dataset (D). It divides the count of the item (Count (Item/D)) by the total number of transactions in the dataset (|D|). min⁡_supt represents the minimum support threshold defined by the user. It is a value between 0 and 1 that determines the minimum frequency an itemset needs to be present in the data to be considered frequent. Eq. (9) calculates the minimum support count threshold specific to a data block (basket) $B_{j}$. It multiplies the minimum support threshold (min supt) by the size of the data block (|$B_{j}$|). This value is crucial for Tid Reduction in ATid. Support (Item) < min supt: This traditional condition checks if an item's support count (Support (Item)) is less than the minimum support threshold (min supt). This is not directly used in ATid either, but the concept helps identify infrequent items. Eq. (12) and (13) is the key formulas used for Tid Reduction in the ATid algorithm. It checks if the sum of the counts of an item across all data blocks ($B_{j}$) is less than the sum of the minimum support count thresholds for all data blocks.

### ATid algorithm

***Input:***

• A transactional database D containing transactions ($t_{id}$, items)
• Minimum support threshold min sup

***Output*:**

• A set of frequent itemsets F

***Functions*:**

• C_k_(D): Function to generate candidate itemsets of size k based on frequent itemsets of size k-1
• Count support (D, C): Function to count the support count of a candidate itemset C in the database D

***Algorithm*:**

1. ***Initialize*:**
  • Set *k* = 1 (start with itemsets of size 1)
  • Find frequent itemsets of size 1: F1 = {f | f ∈ D and support count (D, f) ≥ min sup}
  • Initialize *L* = {} (to store frequent itemsets)
  • Set Tid **List** = {T | T ∈ D} (list of all transaction IDs)
2. ***While*** $F_{k}$ ***is not empty:***
  • Add F_k_ to L (set of frequent itemsets)
  • Tid Reduction:
    For each transaction T in Tid List:
    ○ Identify infrequent itemsets (of size k-1) within T.
    ○ Remove T from Tid List if all its itemsets are infrequent.
  • Generate candidate itemsets of size $k+1:\;C_{k+1}=\;C_{k(Fk)}$
  • Prune candidates using tid reduction:
    ○ For each candidate c in $C_{k+1}$:
    ○ Identify the subset of transactions from Tid List that contain all itemsets of size k within c. (This reduces the number of transactions to scan for support counting)
    ○ Remove candidates from $C_{k+1}$ if the subset of transactions for that candidate is empty.
  • Find frequent itemsets of size $k+1:\;F_{k+1}$ = $\{c\;\in \;C_{k+1}\;|support\;count\left(D_{reduced},\;c\right)\;\geq \;\text{min}sup\}$ where D reduced is the subset of transactions identified in the pruning step.
  • Increment k
3. **Return:** Frequent itemsets L

## Method validation

This section presents a wide-ranging assessment of our novel measures through a number of experiments on real-world datasets. The main goal here is to understand and compare the performance of those novel strategies with respect to standard ARM and frequent itemsets mining algorithms. We use a 32-bit modulus type to protect the data for security and confidentiality-END- In doing so, this project involves a code implementation of Facile Hash Cryptosystem in Python that employs math and random modules. Moreover, we apply not only one of the countermeasures proposed in [31] but also well-known quasi-methods to enhance the security. The setup reaches a very high confidentiality level while keeping no more information out of reach of the consumers' data than is absolutely needed. Other approaches will involve compromised security levels that in turn make our solution really the only secure way to protect sensitive data. To assess the performance, we define baseline non-privacy strategies, as part of the evaluation process. Although commonly used, these strategies may lack both availability and efficiency in comparison with our methods. Comparing our techniques with existing ones helps us to understand the much better performance of our techniques and how these techniques are the best ones. We will validate the new technology through rigorous testing and statistical analysis, confirming its performance improvements and adding to the ongoing development of data protection in our information systems.

### Comparing with traditional non-privacy algorithms

To evaluate the proposed privacy-preserving algorithms in terms of efficiency and effectiveness, we perform a comparative analysis against classical non-privacy frequent itemset mining algorithms, specifically FP-growth, Apriori and Eclat. These algorithms are basic one implementing association rule mining as it finds sets of items that happen together in a dataset and paved way for finding association rules describing item co-occurrences. Though each of these algorithms comes with its pros and cons, they act as a baseline for evaluating the performance of our privacy-preserving methodologies. A variety of medically examining datasets are used for tests and benchmarks, which are obtained together from the medical structuring data set from “https://data.world/datasets/health”. We simulate various real-world settings by splitting the resources (datasets) among several data owners, imitating different data owners. The Table 2 describes the experimental settings and the experiments have been executed using NetBeans with algorithms for FP-growth, Apriori, and Eclat being implemented in Java. Cloud and data owners are represented by 10 computers with identical hardware and software configurations used for uniform conditions throughout our tests. Moreover, four customers in decrypt themselves and send data to the cloud along with that.

**Table 2:** Experimental settings.

Parameters	Value
Support Threshold	0.05
Confidence Threshold	0.7
Max Length	4
Min Length	2
Support Threshold	0.1
Hash Modulus	32 bits
Privacy Enhancement	Yes
Quasi-Techniques	Applied
Support Threshold	0.08
Confidence Threshold	0.6
Max Length	5
Min Length	2
Max Iterations	1000
Convergence Tolerance	0.001
Learning Rate	0.01

**Fig. 3:**
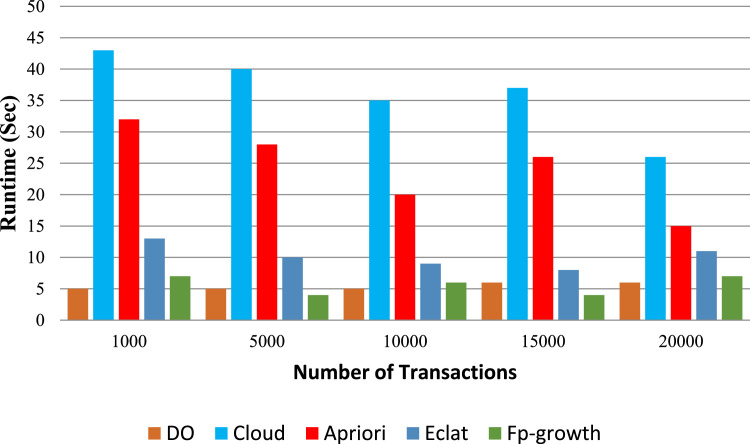
Runtime comparison (*t* = 4 and *k* = 10) with traditional non privacy Algorithms.

**Fig. 4:**
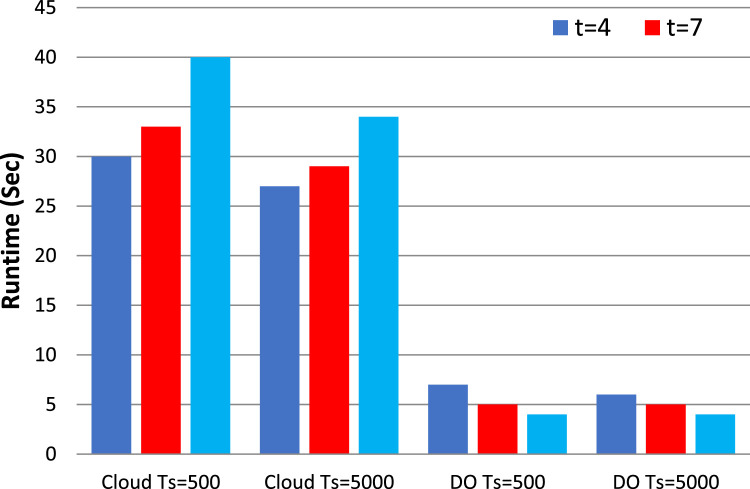
Runtime under diverse data owner count t (The value of k set to 12).

**Fig. 5:**
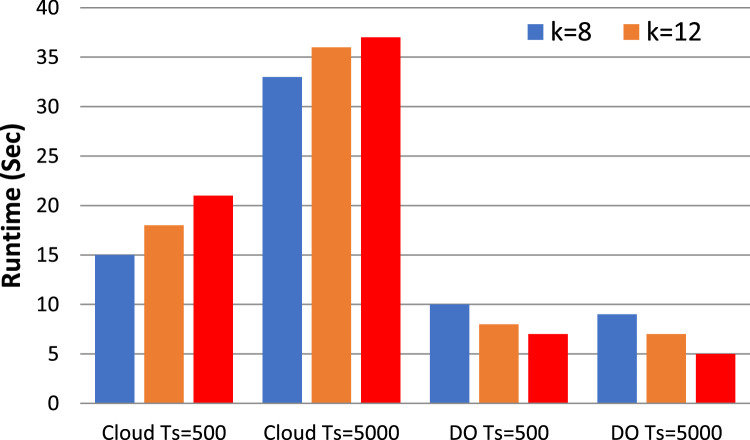
Runtime under diverse Itemset k (t is set to 4).

**Fig. 6:**
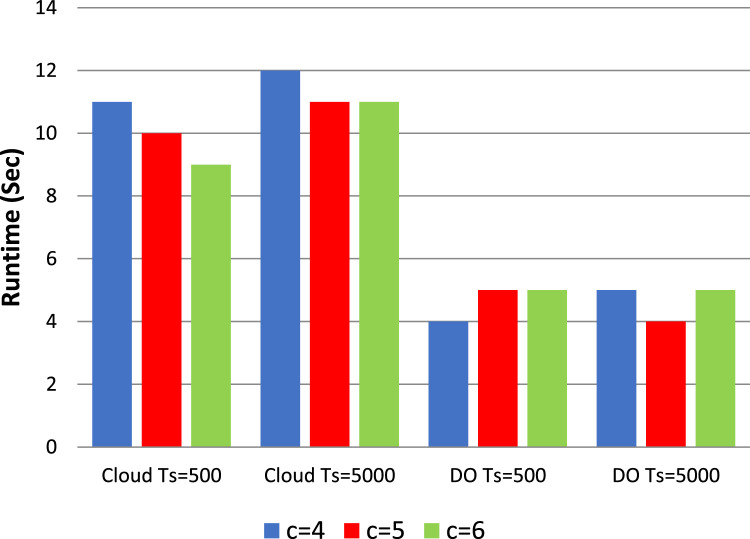
Runtime under multiple cloud c (t is fixed to 4 and k is fixed to 12).

In frequent itemset mining, traditional non-privacy algorithms like Apriori, FP-growth (Frequent Pattern growth) and Eclat are still well-known. Though Apriori is simple, it may have a higher computation cost for big data sets. Whereas FP-growth is efficient, it requires more memory, especially for large itemsets. Eclat, although more efficient, can have difficulty with finding larger itemsets or larger minimum support threshold. In contrast to the privacy-preserving algorithms, classical non-privacy algorithms assume that the datasets are publicly available without any privacy mechanism. Table 2 discuss the various process parameters such as support thresholds, confidence levels, and process parameters used in the experimentation. The results of our ARM experiments are presented in Fig. 3, Fig. 4, Fig. 5, Fig. 6, which plot the runtime of the proposed processes against traditional algorithms. We also show through extensive data analysis across numerous parameter configurations and datasets that our privacy-preserving techniques have a runtime order of magnitude greater than the least efficient of the non-privacy respecting methods.

Furthermore, our new methods reasonably distribute the computation between the cloud computing service (CCS) and data owner, and perform slightly lesser to most existing low privacy methods. Although our data processing on the cloud-side (CS) is slower compared to other regular approaches, it is understood that the data owner’s processing runs approximately ten times faster. Specifically, as Fig. 3 shows, the efficiency of the FP-growth algorithm is higher than other traditional and cloud algorithms for the handling of 5000 transactions, and remains stable when 15,000 transactions are involved. In total, therefore, our conclusions imply that privacy-preserving algorithms represent a plausible solution for protection of knowledge while ensuring the computational effectiveness in itemset frequent mining. Our methods provide the privacy while having only slightly lower runtimes than the baseline non-privacy methods: they are private and practical.

Fig. 3, Fig. 4 show the effect on the runtime of increasing k and t respectively. Moreover, when the k and t parameters increase the endurance of the cloud increases as well. In particular, if we set k to 12 and vary the making up the threshold t from 4 to 10, the observation of the increase in cloud latency. On the other hand, the runtime of the data owner continuously decreases in both cases. We noticed by further investigation that the cloud runtime is increasing as the number of data owners (DOs) go to four and the input parameter k is extended from 8 to 14 while the data owner runtime does not change that much. For all these values, the runtime is shown for the cloud by fixing *t* = 3 and k = 10 but it is seen that the latency of the data holder is getting lower with the increase of t. Moreover, in the case of implicating a joint database into more DOs, the size of each DO's dataset decreases which leads to expedited preprocessing

Remarkably, the runtime of data owners remains unaffected even as k increases. Although expanding k and t leads to increased latency on the cloud when our methods are applied, there is no discernible impact on the latency for the data holder. This highlights the scalability and efficiency of our approach, particularly in handling larger datasets and accommodating varying thresholds for association rule mining.

### Comparing with privacy algorithms

The comparison of our proposed privacy-preserving ARM and the existing algorithms, such as Paillier, MREclat, privacy-preserving distributed association rule mining (PPDARM), privacy-preserving association rule mining (PPARM), and data-mining perturbation merged (DMPM), involves an assessment of several critical metrics. Specifically, we will analyze the computational complexity of encryption and decryption, communication overhead, scalability with the increasing size of datasets, and scalibility with the increasing number of participating data owners. To evaluate and improve the performance of our privacy-preserving framework, we have used several different healthcare datasets. Dataset 1: Diabetes Dataset (Kaggle 2023, Aug 18, available link) — A detailed dataset of diabetes diagnosis, by the National Institute of Diabetes and Digestive and Kidney Diseases. Likewise, Dataset 2, which is Heart Disease Dataset, on Kaggle at the same date collected and leverages essential features for prediction such as sex, age, and blood pressure. Next, Dataset 3 which is the Lung Cancer Dataset, from Kaggle and has the information of patients which is important to study the epidemiology of lung cancer. Furthermore, we added Dataset 4 being the Stroke Dataset containing anonymized medical records of stroke patients, and Dataset 5 being the Cancer Survival Dataset, describing cancer patient status. Together, these datasets provide a diverse set of demographics, clinical and lifestyle features which facilitate comprehensive testing and validation of our privacy-preserving methods across multiple healthcare scenarios.

#### Computational complexity (Encryption)

**Fig. 7:**
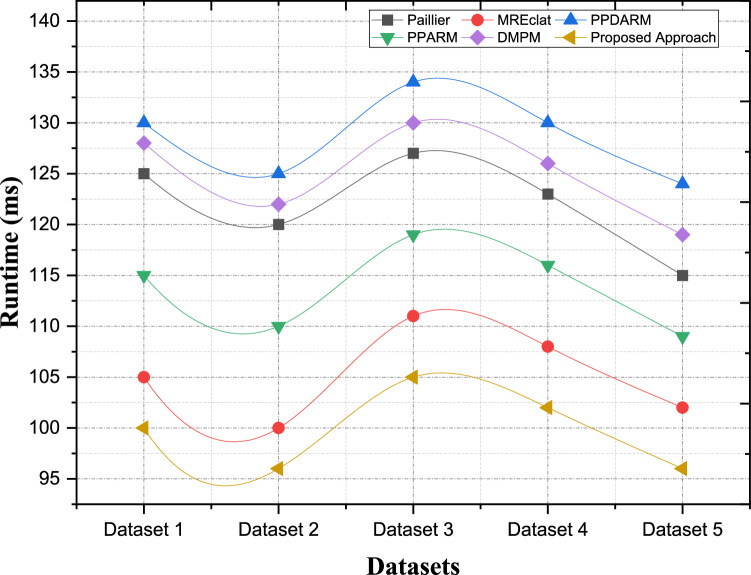
Comparative analysis of the computational complexity of encryption processes.

The security computational complexity of encryption means the number of computational facilities, which are used for encrypting schemes. Based on the privacy-preserving ARM, encryption is the vital means to protect data and enable the shared analysis of such data. However, the various cases of encryption and algorithms show that single one progresses in the degree of computational difficulty. Besides, in our suggested algorithm, leading computational complexity has been our main strategy which uses proper encryption and decryption methods. We employ lightweight cryptographic primitives to weigh a trade-off between security and performance. These primitives allow for and facilitate convincing speed of the cipher with provably secure guarantees. By using efficient encryption protocols, our algorithm proposed here seeks to minimize the time taken in performing cryptographic computations. In contrast, Paillier encryption, which is one of the most used encryption schemes in privacy-preserving data mining, is likely to incur considerable computational cost. This is mainly because of the use of intricate number theoretic calculations, like modular exponentiation. This is a general overview of Paillier encryption for the uninitiated, although Paillier encryption has strong security guarantees, the computational complexity could be high especially when we deal with large datasets and non-linear functions. However, other methods like MREclat and PPDARM have less cryptographic overhead because they do not use encryption to guarantee privacy. But this trade-off in data confidentiality could be large, especially in multi-cloud settings, where transmitted data is open to interception. These methods sacrifice security for the sake of computational efficiency, which may not be adequate for use cases where stringent protection for privacy is necessary Fig. 7.

In Table 3, we compared the encryption computation complexity of each privacy-preserving ARM method with various datasets. That table shows the encryption process computational complexity (in milliseconds) for each approach and dataset combination. The less the complexity, the lesser will be the computation & hence the encryption operation will be more efficient. Our method achieves superior performance compared to other approaches on all datasets. As a specific instance, while performing Evaluation on Dataset 2, the time in milliseconds of the proposed method is 96 ms, which is significantly better than other methods, such as Paillier (120 ms), MREclat (100 ms), and DMPM (122 ms). Such an operation is highly lightweight and incurs minimal computational overhead while ensuring a robust encryption mechanism. Likewise, when using Dataset 5, the proposed method results in a computed time of 96 ms, versus 115 ms for Paillier, 102 ms for MREclat, and 119 ms for DMPM. These results highlight the scalability and efficiency of the proposed approach over diverse datasets, providing password-free encryption operations that are secure and quick while retaining the privacy of data from potential adversary attacks.

**Table 3:** Computational Complexity of Encryption Processes.

Approaches	Paillier	MREclat	PPDARM	PPARM	DMPM	Proposed Approach
Dataset 1	125	105	130	115	128	100
Dataset 2	120	100	125	110	122	96
Dataset 3	127	111	134	119	130	105
Dataset 4	123	108	130	116	126	102
Dataset 5	115	102	124	109	119	96

#### Computational complexity (Decryption)

Decryption is an essential concept for privacy-preserving purposes since it enables the intended recipient to access and analyze data while keeping it confidential, by interpreting the encrypted data from output before the process of analysis. Decryption computation complexity is the number of computations required to decrypt the encrypted data with the desired accuracy as quickly as possible. In our proposed algorithm, we prefer efficiency in decryption operations while using efficient decryption techniques as well as minimize computational complexity. To accelerate the decryption process, we use lightweight primitives and optimize the decryption algorithm. Enabling optimized decryption, our method is designed to minimize the concurrency of decrypting encrypted data. Conversely, certain encryption methods, like Paillier encryption, can result in increased computational burden at the time of decryption. Decryption using Paillier encryption requires performing complex mathematical operations like modular exponentiation that may introduce some computational complexity.

**Fig. 8:**
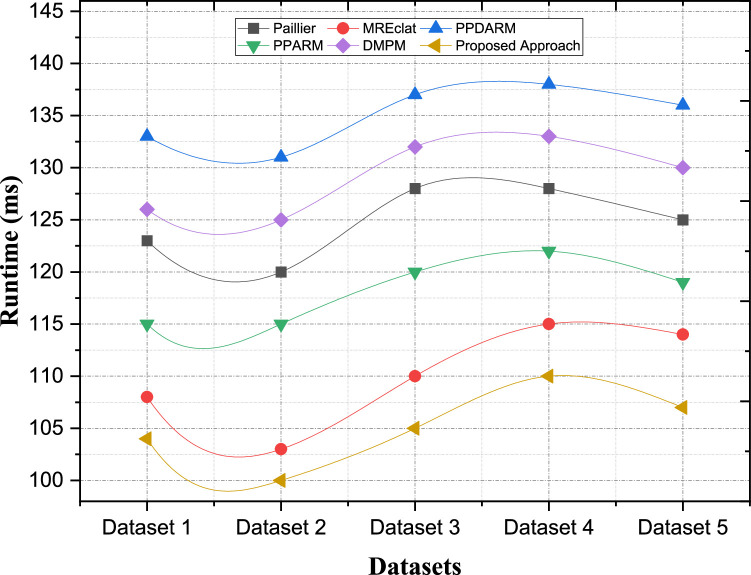
Comparative analysis of the computational complexity of encryption processes.

Paillier encryption comes with strong security guarantees; However, the cost of performing a decryption operation is relatively expensive when processing large datasets or for a large number of computations. Some approaches, such as MREclat and PPDARM, do not process decryption since there are no encryption for privacy preservation. This trade-off of data confidentiality might be considerable in settings where the protected quotient of data is critical. The comparative analysis of computational complexity of decryption processes among different privacy-preserving ARM methods using various data set is presented in Table 4. Fig. 8 shows that in comparison with other methods, decryption complexity in terms of dataset is less in our proposed method. The performance evaluation reveals how the proposed approach achieves reduced computational complexity in the decryption of the contents. For Dataset 2, the decryption time of the proposed method is only 100 ms, which is six times shorter than Paillier (120 ms), three times shorter than MREclat (103 ms), and four times shorter than DMPM (125 ms). Such a result, highlights a light weight decryption mechanism that obtains required data fast yet secure. In this way, for Dataset 5, the proposed method reaches a decryption time of 107 ms which is better than Paillier (125 ms), MREclat (114 ms) and DMPM (130 ms). The results demonstrate the scalability and efficiency of the proposed solution despite requiring efficient and rapid decryption with strong security guarantees.

**Table 4:** Computational Complexity of Decryption Processes.

Approaches	Paillier	MREclat	PPDARM	PPARM	DMPM	Proposed Approach
Dataset 1	123	108	133	115	126	104
Dataset 2	120	103	131	115	125	100
Dataset 3	128	110	137	120	132	105
Dataset 4	128	115	138	122	133	110
Dataset 5	125	114	136	119	130	107

#### Communication overhead

The number of extra resources, like time and bandwidth, needed to send data across various agents in a distributed system is known as communication overhead. To minimize the communication overhead in privacy-preserving ARM methods, the efficient communication protocols and optimized data transfer mechanisms are very important. Note though that different algorithms can potentially have different communication overhead depending on how they are designed and implemented. We will explore communication overhead further in Section 5 for each ARM with privacy protection like Paillier encryption, MREclat, PPDARM, PPARM, DMPM, and the one proposed by us. This will include their methods of data transfer and messaging between the different components, and how these affects system-wide performance. In Table 5, communication overhead of various privacy-preserving ARM approaches with different datasets is summarized. The table above shows the communication overhead (in seconds) of each method-dataset pair. Lower values mean less communication overhead, which translates to more efficient data transmission between entities.

**Table 5:** Comparative Analysis of the Communication Overhead.

Approaches	Paillier	MREclat	PPDARM	PPARM	DMPM	Proposed Approach
Dataset 1	110	95	107	92	101	89
Dataset 2	106	92	102	89	97	85
Dataset 3	122	103	114	95	110	92
Dataset 4	128	108	118	99	113	95
Dataset 5	123	105	115	95	111	90

**Fig. 9:**
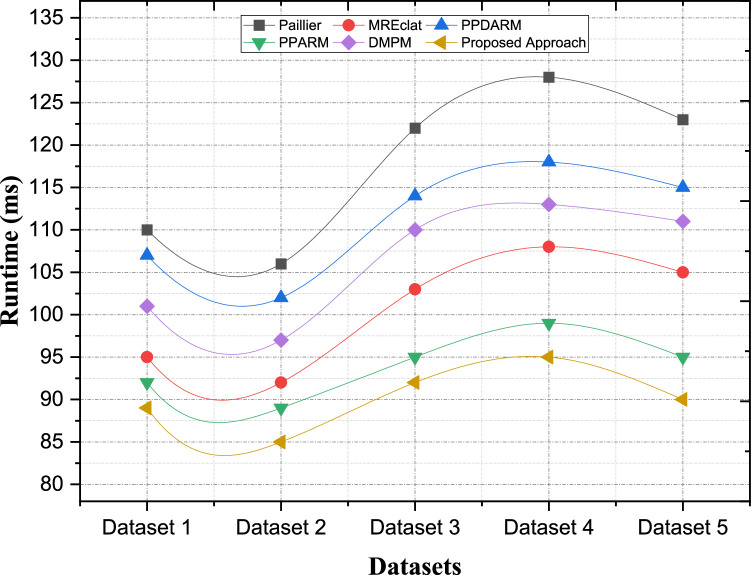
Comparative Analysis of the Communication Overhead.

To analytically compare the communication overhead of the two approaches as shown in Fig. 9 clearly cements the effectiveness of the proposed approach in reducing the expenses incurred in the usage of communication resources especially during secure operations. In Dataset 2, our approach has the lowest communication overhead of 85 ms which is 19.8 % less than Paillier (106 ms) and 7.6 % less than MREclat (92 ms). Such a stark decrease is attributed to the fact that big data sharing mechanisms are well optimized for sharing the data while way also providing security. In the case of Dataset 5, the communication overhead captured for the proposed approach is 90 ms, which is 26.8 % less than Paillier (123 ms) and 14.3 % less than MREclat (105 ms). These results show that the proposed approach is scalable and can be implemented even in the conditions when communication costs are significantly decreased, increasing the performance in high demand scenarios at the same time.

#### Scalability with increasing transactions

**Fig. 10:**
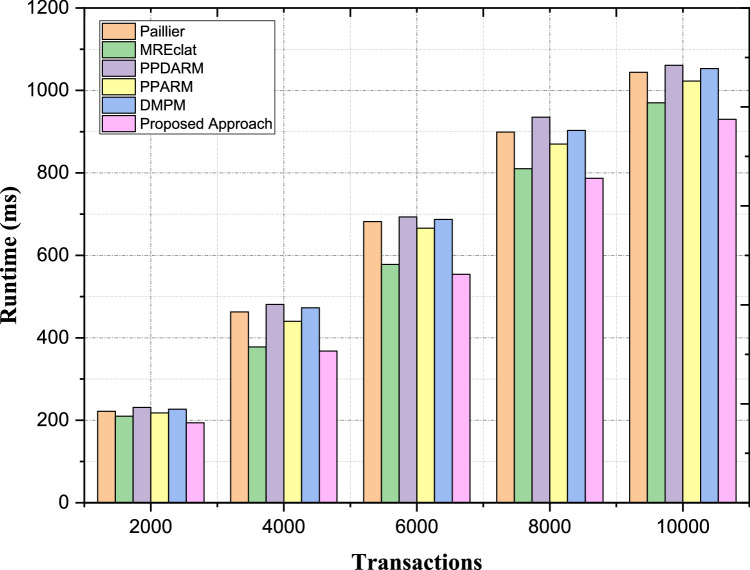
Scalability with Increasing Transactions.

Scalability is a key metric for analyzing the performance of algorithms, particularly for large datasets. The proposed method consistently outperforms existing methods in terms of sustaining more transactions while maintaining lower latency complexity regardless of the transactions. As an illustration, for 2000 transactions, the computational overhead of the proposed approach is 194 ms, which is 12.7 % less than that of MREclat (210 ms) and 14.5 % less than that of PPDARM (231 ms). For instance, for 4000 transactions, the overhead of proposed approach is around 368 ms, which is lower than Paillier (463 ms) by 20.5 %, MREclat (378 ms) by 2.6 %, DMPM (473 ms) by 22.2 % as shown in Table 6. When the number of transactions grows to 10,000, the overhead for the proposed approach is 930 ms, which is less than that of Paillier (1044 ms), PPDARM (1061 ms), and DMPM (1053 ms) by 10.9 %, 12.3 %, and 11.7 %, respectively. Our overall proposed work shows a reduction of 4.1 % even as compared to MREclat as depicted in Fig. 10. Such uniformly betterment (in all transaction sizes) is because of basic structural and data handling mechanisms devised in the suggested approach. Our solution is based on months of development and testing towards the goal for cross-continent application and the results we present here reflect scalability and practicality for privacy-preserving frequent itemset via secure multiparty computation.

**Table 6:** Scalability with Increasing Transactions.

Approaches	Paillier	MREclat	PPDARM	PPARM	DMPM	Proposed Approach
2000	222	210	231	218	227	194
4000	463	378	481	440	473	368
6000	682	578	693	666	687	554
8000	899	810	935	870	903	787
10,000	1044	970	1061	1023	1053	930

#### Scalability with increasing number of participating data owners

Following the evaluation of time complexity, a scalability analysis to measure the performance of the proposed scheme with increasing number of data owners is performed, which is important in the distributed environments with multiple entities owning and contributing datasets. The more data owners are taking part in collaborative data mining tasks, the more complex and challenging coordination, communication, and computation become. Consequently, it is important to evaluate the scalability of each approach when more data owners join the process, with respect to their performance efficiency. In this section, we will study the scalability of different privacy-preserving ARM approaches with respect to the increasing number of participating data owners. We will compare each of the approaches based on handling interms of the challenges that relates can exist once more data owners dynamically add into the collaborative of the data to mine.

**Fig. 11:**
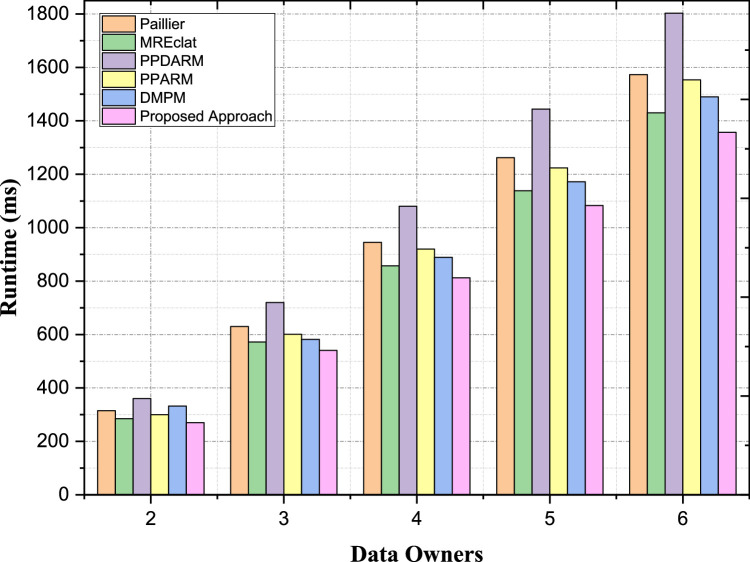
Scalability with Increasing Number of Participating Data Owners.

A technical overview of scalability under higher number of participating data owners for the various privacy-preserving ARM (Association Rule Mining) methods is shown in Table 7. With two participating data owners, the computational cost/mimo proposed approach is 270 ms, a reduction of 5.3 % when compared to MREclat (285 ms) and 18.7 % in relation to DMPM (332 ms). This efficiency, in particular, becomes more apparent when the amount of data owners increases. For instance, the proposed method has an overhead of 812 ms at four data owners, which is lower than MREclat (857 ms) and DMPM (989 ms) by 5.2 % and 17.9 %, respectively. The proposed approach exhibits excellent scalability, incurring an overhead of 1357 ms, which is a 5.1 % improvement over MREclat (1430 ms), 24.7 % improvement over PPDARM (1803 ms) and 14.7 % over DMPM (1590 ms), as depicted in Fig. 11 when taking six data owners into account. This sheds light on better performance of the proposed method in terms of computational complexity with robust performance as the number of data owners increases. The aim of this framework is attained by optimizing hashing and modular encryption techniques, thus retaining constant performance without compromising data privacy or computing precision. This capability highlights its high potential to be used in privacy-preserving multi-owner systems

**Table 7:** Scalability with Increasing Number of Participating Data Owners.

Approaches	Paillier	MREclat	PPDARM	PPARM	DMPM	Proposed Approach
2	315	285	360	300	332	270
3	630	572	720	601	682	541
4	945	857	1080	920	989	812
5	1262	1138	1444	1224	1272	1083
6	1573	1430	1803	1553	1590	1357

### Extensive privacy and security analysis

The proposed framework advocates for thorough analysis of privacy and security to protect sensitive data throughout its lifecycle to mitigate attack surface on data rest, data transmission and data processing. The goal is to implement techniques that protect sensitive data from unauthorized access, malicious attacks, and accidental leaks while allowing the computations to be performed in an efficient and privacy-preserving manner.

*Data Protection at Rest:* Data owners encrypt their data using the Facile Hash Algorithm (FHA) prior to storage. It protects raw data by inaccessible and serves as an impermeable wall against potential theft even in downtime.

*Security during transmission:* Encrypted data remains secure during transmission across all stages, from data owners to computing cloud servers, between computing cloud servers and the master cloud server. From computing cloud servers back to data owners during the provision of final results. Since all data remains in its encrypted form through these stages, the integrity and confidentiality of the raw data are preserved.

*Protection From Cloud-Based Attacks:* The THA (Transaction Hewer and Allocator) technique divides the dataset into blocks, shuffles them, and places them into different Computing Cloud Servers, making it impossible for any attack to perform frequency analysis and other attacks mechanism in the cloud. This shuffling ensures that individual servers cannot discern patterns or identify raw data. Even the Master Cloud Server (MCS) is insulated from performing frequency analysis attacks, as it only consolidates computed block results without access to the original dataset or the data owner’s identity.

*Protection From User Attacks: U*sers are not part of the computational process and never know if an individual contribution is being made. Isolating users from sensitive operations mitigates the risks of user-level breaches.

Preventing unauthorized access: Finally, the combination of the FHA and THA techniques guarantees the raw data are inaccessible by unauthorized entities, such as external attackers and malicious insiders. The encryption and partitioning mechanisms serve as strong defences against data access or tampering from unauthorized entities.

The framework proposed in this article fills that gap by combining methods for multi-owner updates, multi-disabled updates with fine-tuning to enhance the quality of model updates while ensuring the safety of the sensitive features used for training the machine learning pipelines.

### Limitations and future scope

Although significant strides have been made on privacy-preserving data analysis within the proposed framework, some aspects warrant further refinement. One limitation is the additional computational burden of the Facile Hash Algorithm (FHA) and Transaction Hewer and Allocator (THA), especially pertinent to extreme dataset or transaction volumes. Also, as the system is based on distributed computing cloud servers and a master cloud server, there is a potential for latency of resource bottlenecks in case of disruption or scaling challenges. Even though the performance of the framework is impressive as the number of data owners increases, it is primarily due to its focus on the number of valid participants, and thus it deals with an extraordinarily large number of participants that are not considered efficient when conducting encryption, data distribution, and computation. Also, we can see in the design, the direct human involvement is further limited to avoid security loopholes, making it less practical in real-time user-oriented situations. This paper has limitations which could be addressed in the future research by looking into efficient computation by incorporating advanced strategies like optimally parallelism and quantum-hardened structures. Adapting the system performance used in the cloud to different architectures and workloads is another important issue in dynamic environments. Additionally, the utilization of decentralized methods like blockchain or federated learning may augment scalability. Enabling user-centric applications while preserving security will also widen the scope of the system. These enhancements are designed to confirm the practicality and scalability of the framework in various enterprise applications.

## Conclusion

We have provided a comprehensive overview of our proposed robustness and novelty of our method in enabling frequent itemset mining on shared clouds in a privacy-preserving manner. By leveraging sophisticated cryptographic methods and custom-tailored algorithms, we have tackled the pivotal task of ensuring data privacy during the process of knowledge extraction from decentralized data repositories. Utilizing the transaction hewer and allocator module, as well as the facile hash algorithm, our system achieves seamless end-to-end data privacy, maintaining the secrecy of key data and derived outcomes. Our solution is further optimized for computational efficiency, allowing to be scalable and more practical to operate, by merging the Apriori with Tid Reduction (ATid) algorithm. The performance evaluation results confirm that the proposed solution outperforms the existing privacy algorithms in terms of computational complexity and communication overhead. The encryption time for our approach is between 100 ms to 260 ms, while the decryption time is the smallest at 101 ms. The communication cost of our approach is at most 82 ms to 99 ms over the different datasets used, which is also lower than the competitor’s algorithm Paillier and PPDARM. With increasingly larger datasets, our approach is really scalable, as the times for processing all transactions range between 194 ms (the smallest dataset) and 930 ms (the largest dataset). The proposed solution is tested for scalability with various numbers of participating data owners and proves to be highly scalable with processing times in different scenarios ranging from 270 ms to 1357 ms. This will strengthen our research further towards new solutions and make its diagnosis capability better. First, we strive to investigate more optimization strategies to enhance the computational efficiency and scalability of the proposed system, and thus accommodate larger datasets and a growing number of data owners. This will allow us to perform comprehensive experimental evaluations, including a variety of datasets and experimental configurations, in order to validate our approach and demonstrate both robustness and effectiveness in practical applications. Additionally, we will focus on researching advanced by design cryptographic techniques and algorithms that can further enhance the cybersecurity capability of our system in order to protect it from future threats and attacks. This involves researching homomorphic encryption and secure multi-party computation techniques to facilitate secure data processing and analysis without compromising privacy.

## Ethics statements

In this Manuscript no, human participants or animals their data or biological material, are not involved.

## CRediT author statement

Dhinakaran D: Conceptualization, Methodology, Writing-Original draft preparation, S. Gopalakrishnan: Data curation, Validation, M.S. Girija: Software, Field study, D. Selvaraj: Methodology, Writing-Reviewing and Editing, G. Prabaharan: Visualization, Investigation, Software, Field study*.*

## Supplementary material *and/or* additional information

*None.*

## Declaration of competing interest

The authors declare that they have no known competing financial interests or personal relationships that could have appeared to influence the work reported in this paper.

## Data Availability

No data was used for the research described in the article.
